# Supplementary figures and images for: 3D CNN for neuropsychiatry: Predicting Autism with interpretable Deep Learning applied to minimally preprocessed structural MRI data
Source: PLoS One. 2024 Oct 21;19(10):e0276832. doi: 10.1371/journal.pone.0276832 (PMC11493284; doi:10.1371/journal.pone.0276832)

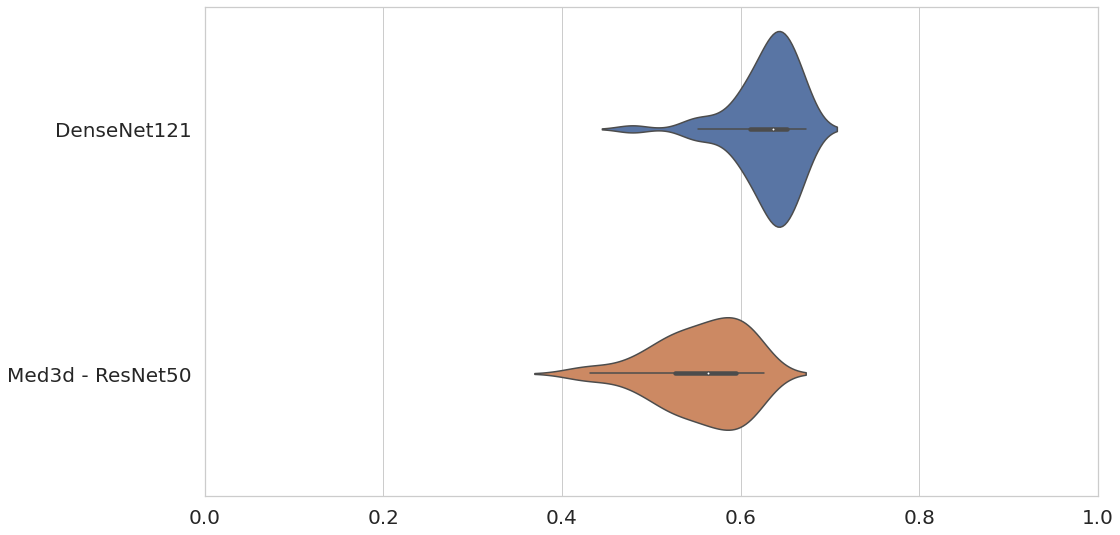

Supplement: S1 Fig — DenseNet121 tended to have higher accuracy on the validation set than Med3d-ResNet50. (PNG) [file pone.0276832.s021.png]

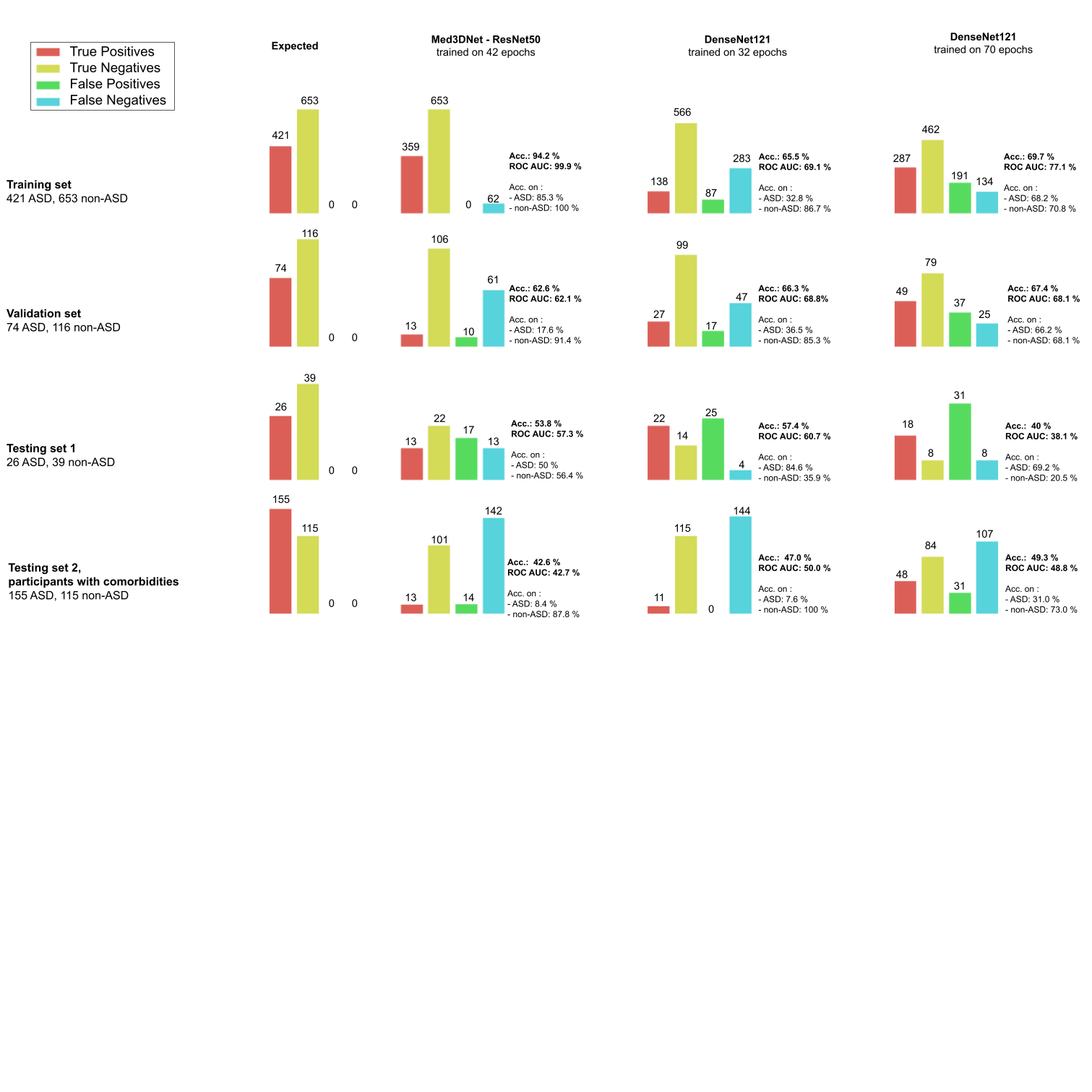

Supplement: S2 Fig — (PNG) [file pone.0276832.s022.png]

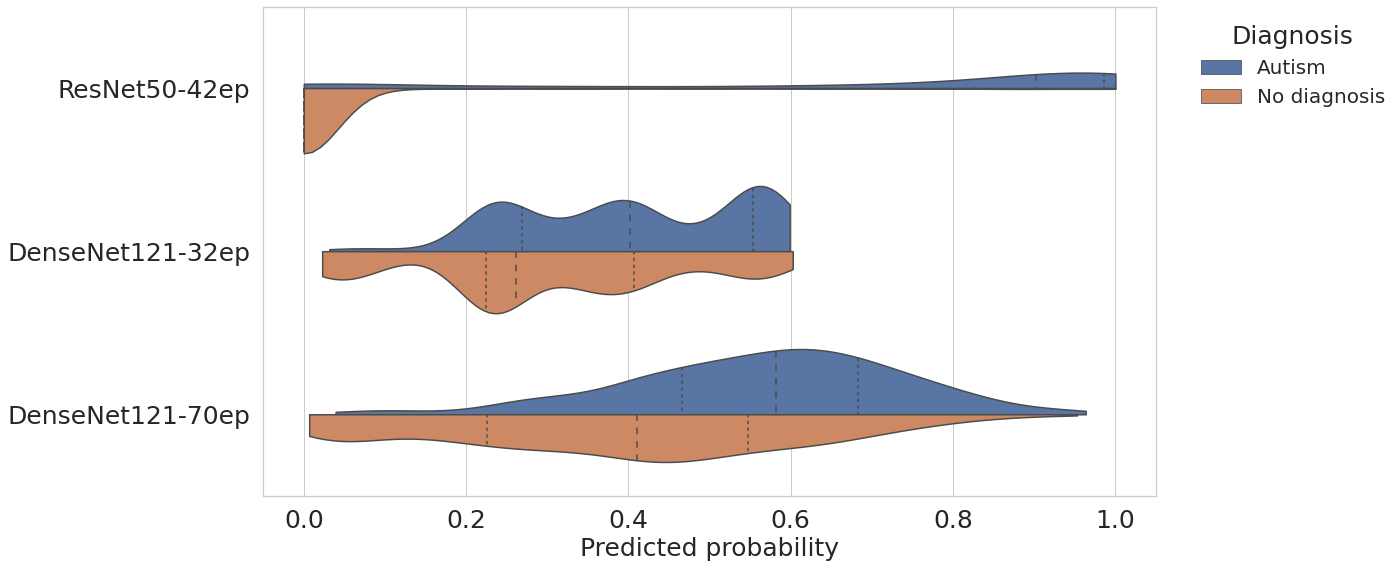

Supplement: S3 Fig — (PNG) [file pone.0276832.s023.png]

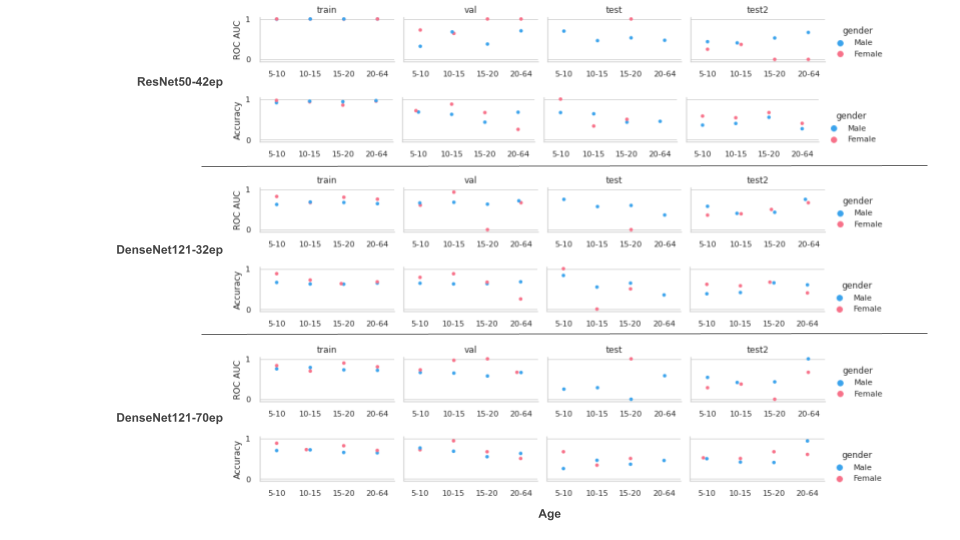

Supplement: S4 Fig — (PNG) [file pone.0276832.s024.png]

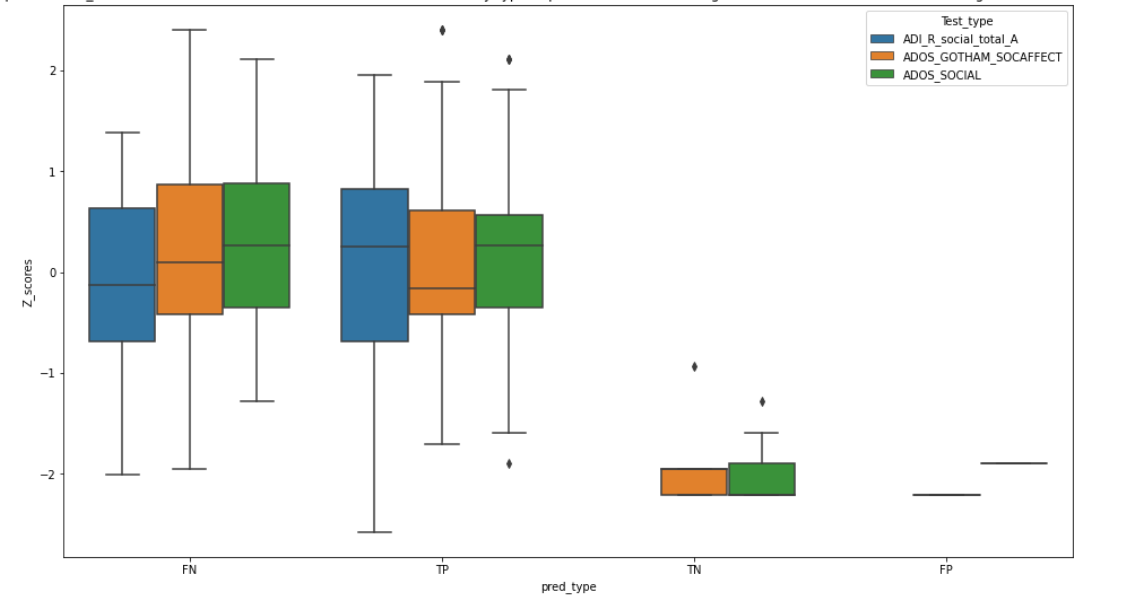

Supplement: S5 Fig — (PNG) [file pone.0276832.s025.png]

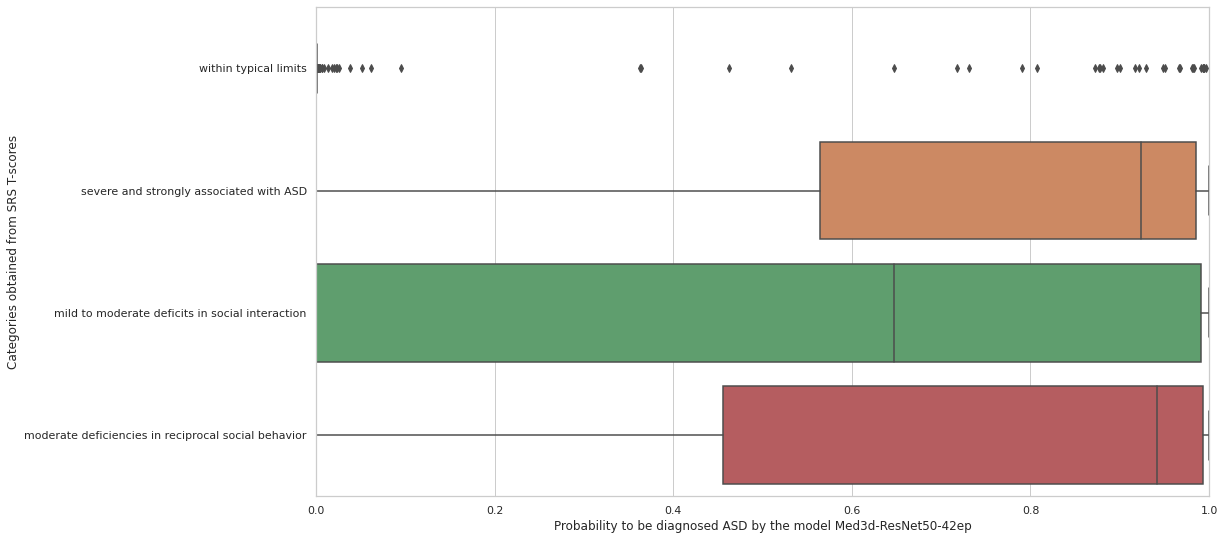

Supplement: S6 Fig — (PNG) [file pone.0276832.s026.png]

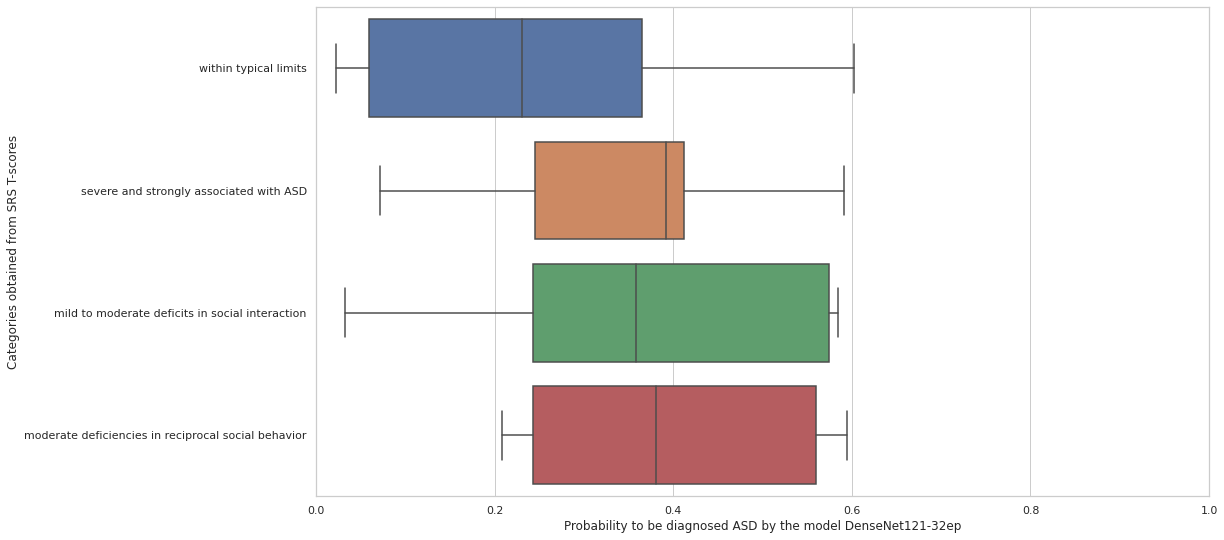

Supplement: S7 Fig — (PNG) [file pone.0276832.s027.png]

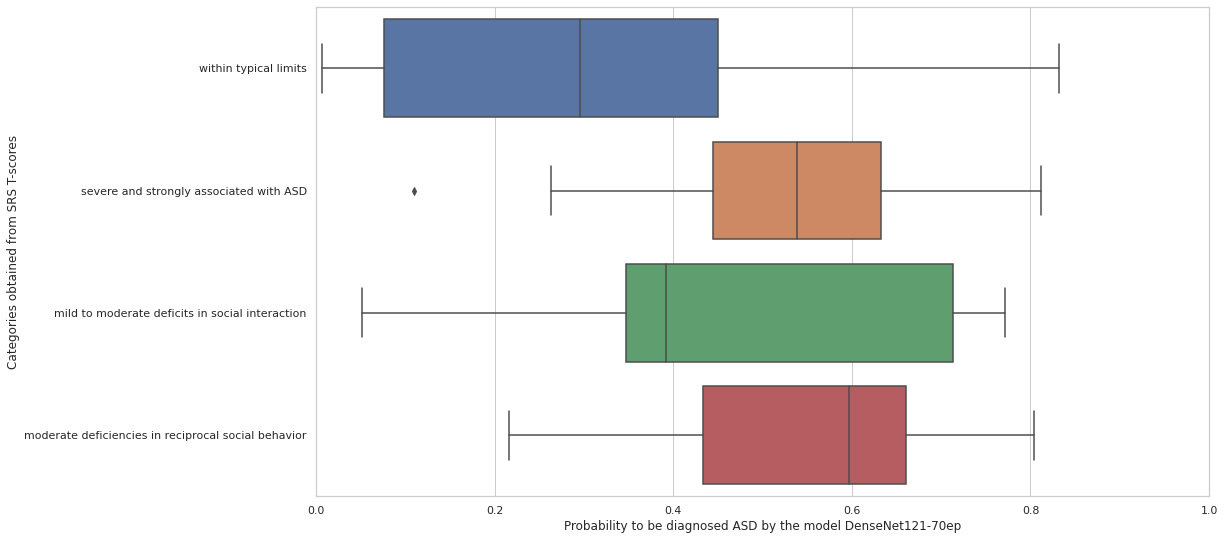

Supplement: S8 Fig — (PNG) [file pone.0276832.s028.png]

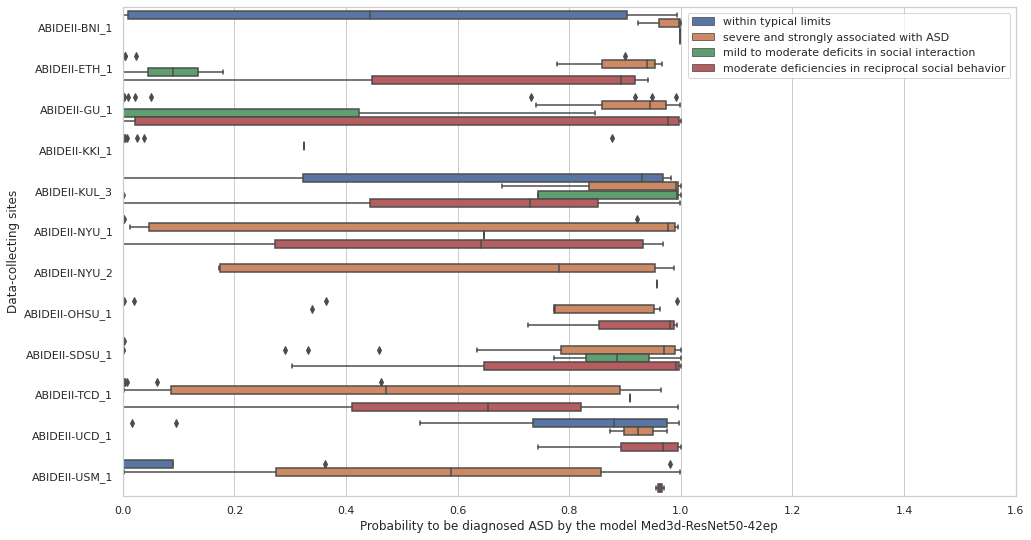

Supplement: S9 Fig — (PNG) [file pone.0276832.s029.png]

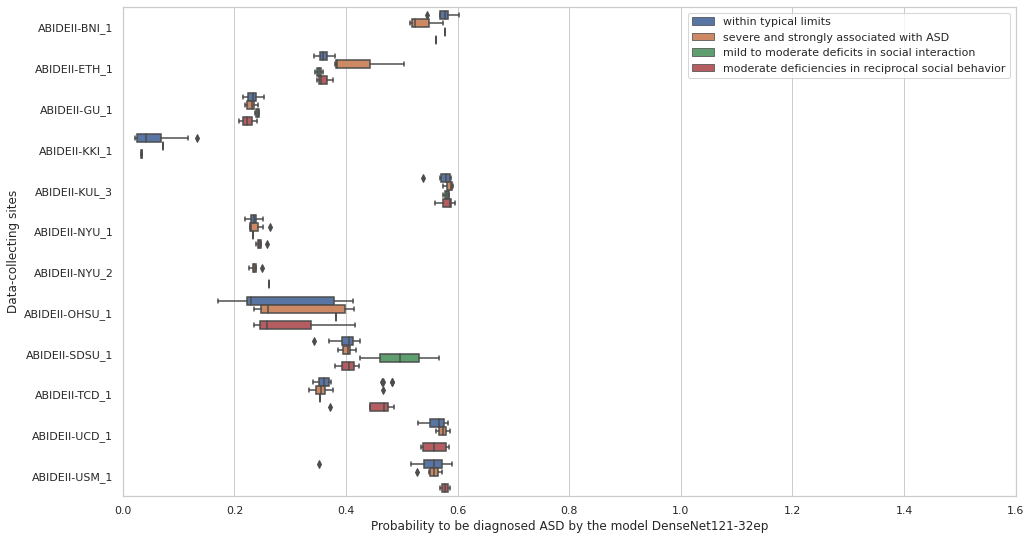

Supplement: S10 Fig — (PNG) [file pone.0276832.s030.png]

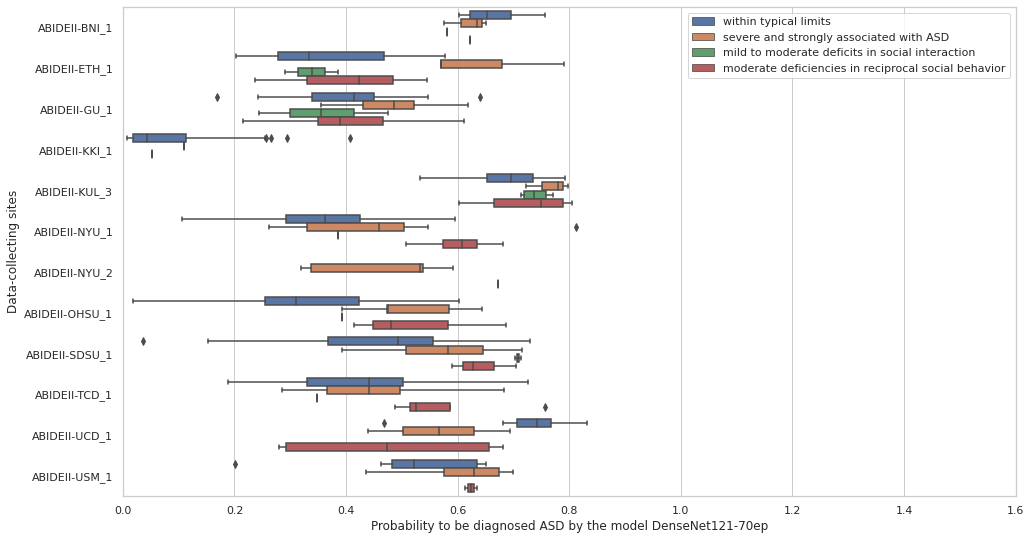

Supplement: S11 Fig — (PNG) [file pone.0276832.s031.png]

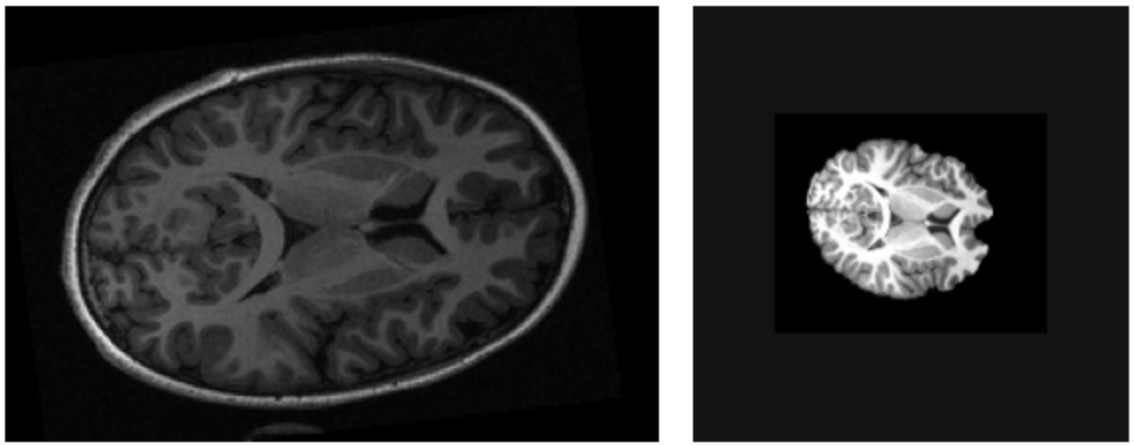

Supplement: S12 Fig — Original voxel size was 1.2mm*1mm*1mm. Original image size was 160*240*256. (PNG) [file pone.0276832.s032.png]

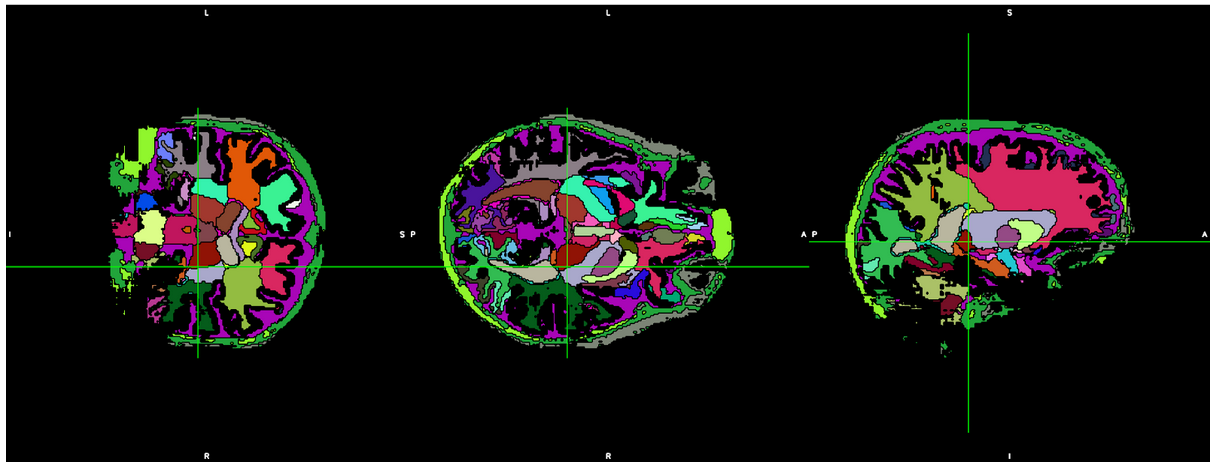

Supplement: S13 Fig — (PNG) [file pone.0276832.s033.png]
