# Supplementary material for: 3D CNN for neuropsychiatry: Predicting Autism with interpretable Deep Learning applied to minimally preprocessed structural MRI data
Source: PLoS One. 2024 Oct 21;19(10):e0276832. doi: 10.1371/journal.pone.0276832 (PMC11493284; doi:10.1371/journal.pone.0276832)
Supplement: Attachment — Submitted filename: Response to Reviewers.docx [file pone.0276832.s034.docx]

PONE-D-22-28394

Towards 3D Deep Learning for neuropsychiatry: predicting Autism diagnosis using an interpretable Deep Learning pipeline applied to minimally processed structural MRI data

5946 Words, 2 Figures and 1 Tables

PLOS ONE

Dear Dr. Garcia,

Thank you for submitting your manuscript to PLOS ONE. After careful consideration, we feel that it has merit but does not fully meet PLOS ONE’s publication criteria as it currently stands. Therefore, we invite you to submit a revised version of the manuscript that addresses the points raised during the review process.

Please submit your revised manuscript by Aug 13 2023 11:59PM. If you will need more time than this to complete your revisions, please reply to this message or contact the journal office at plosone@plos.org. When you're ready to submit your revision, log on to<https://www.editorialmanager.com/pone/> and select the 'Submissions Needing Revision' folder to locate your manuscript file.

Please include the following items when submitting your revised manuscript:

- A rebuttal letter that responds to each point raised by the academic editor and reviewer(s). You should upload this letter as a separate file labeled 'Response to Reviewers'.
- A marked-up copy of your manuscript that highlights changes made to the original version. You should upload this as a separate file labeled 'Revised Manuscript with Track Changes'.
- An unmarked version of your revised paper without tracked changes. You should upload this as a separate file labeled 'Manuscript'.

If you would like to make changes to your financial disclosure, please include your updated statement in your cover letter. Guidelines for resubmitting your figure files are available below the reviewer comments at the end of this letter.

 If applicable, we recommend that you deposit your laboratory protocols in [protocols.io](http://protocols.io) to enhance the reproducibility of your results. Protocols.io assigns your protocol its own identifier (DOI) so that it can be cited independently in the future. For instructions see:<https://journals.plos.org/plosone/s/submission-guidelines#loc-laboratory-protocols>. Additionally, PLOS ONE offers an option for publishing peer-reviewed Lab Protocol articles, which describe protocols hosted on [protocols.io](http://protocols.io). Read more information on sharing protocols at<https://plos.org/protocols?utm_medium=editorial-email&utm_source=authorletters&utm_campaign=protocols>.

 We look forward to receiving your revised manuscript.

Kind regards,

Francesca Benuzzi, Ph.D.
 Academic Editor
 PLOS ONE

Journal Requirements:

When submitting your revision, we need you to address these additional requirements.

1. Please ensure that your manuscript meets PLOS ONE's style requirements, including those for file naming. The PLOS ONE style templates can be found at
<https://journals.plos.org/plosone/s/file?id=wjVg/PLOSOne_formatting_sample_main_body.pdf> and
<https://journals.plos.org/plosone/s/file?id=ba62/PLOSOne_formatting_sample_title_authors_affiliations.pdf>

2. Our internal editors have looked over your manuscript and determined that it is within the scope of our Reproducibility and Replicability in Neuroscience and Mental Health Research Call for Papers. The Collection will encompass a diverse and interdisciplinary set of protocols and research articles adhering to transparent and reproducible reporting practices in the areas of clinical psychology, psychiatry, mental health, and neuroscience. Additional information can be found on our announcement page:<https://collections.plos.org/call-for-papers/reproducibility-and-replicability-in-neuroscience-and-mental-health-research/>. If you would like your manuscript to be considered for this collection, please let us know in your cover letter and we will ensure that your paper is treated as if you were responding to this call. If you would prefer to remove your manuscript from collection consideration, please specify this in the cover letter.

3. Please amend either the title on the online submission form (via Edit Submission) or the title in the manuscript so that they are identical.

4. Please include your full ethics statement in the ‘Methods’ section of your manuscript file. In your statement, please include the full name of the IRB or ethics committee who approved or waived your study, as well as whether or not you obtained informed written or verbal consent. If consent was waived for your study, please include this information in your statement as well.

We have included a new section 2.6 Ethics statement (p15).

5. Please include captions for your Supporting Information files at the end of your manuscript, and update any in-text citations to match accordingly. Please see our Supporting Information guidelines for more information:<http://journals.plos.org/plosone/s/supporting-information>.

Additional Editor Comments:

 Dear authors,

 I have sent your paper to three reviewers, and two of them have recommended major revisions but, I firmly believe that the issues raised can be addressed.
 In particular, Reviewer 2 recommends greater transparency and accuracy in describing the methods, so that even those who are not familiar with the subject matter can fully understand the methodological aspects of the work.

 Reviewer 3, on the other hand, emphasizes the need for a thorough revision of the text and figures.

 Best regards,


 [Note: HTML markup is below. Please do not edit.]

 Reviewers' comments:

 Reviewer's Responses to Questions

**Comments to the Author**

 1. Is the manuscript technically sound, and do the data support the conclusions?

 The manuscript must describe a technically sound piece of scientific research with data that supports the conclusions. Experiments must have been conducted rigorously, with appropriate controls, replication, and sample sizes. The conclusions must be drawn appropriately based on the data presented.

Reviewer #1: No

Reviewer #2: Yes

Reviewer #3: Yes

2. Has the statistical analysis been performed appropriately and rigorously?

Reviewer #1: No

Reviewer #2: I Don't Know

Reviewer #3: Yes

3. Have the authors made all data underlying the findings in their manuscript fully available?

 The [PLOS Data policy](http://www.plosone.org/static/policies.action#sharing) requires authors to make all data underlying the findings described in their manuscript fully available without restriction, with rare exception (please refer to the Data Availability Statement in the manuscript PDF file). The data should be provided as part of the manuscript or its supporting information, or deposited to a public repository. For example, in addition to summary statistics, the data points behind means, medians and variance measures should be available. If there are restrictions on publicly sharing data—e.g. participant privacy or use of data from a third party—those must be specified.

Reviewer #1: Yes

Reviewer #2: Yes

Reviewer #3: Yes

4. Is the manuscript presented in an intelligible fashion and written in standard English?

 PLOS ONE does not copyedit accepted manuscripts, so the language in submitted articles must be clear, correct, and unambiguous. Any typographical or grammatical errors should be corrected at revision, so please note any specific errors here.

Reviewer #1: No

Reviewer #2: Yes

Reviewer #3: Yes

5. Review Comments to the Author

 Please use the space provided to explain your answers to the questions above. You may also include additional comments for the author, including concerns about dual publication, research ethics, or publication ethics. (Please upload your review as an attachment if it exceeds 20,000 characters)

Reviewer #1: The authors propose an AI method, based on 3D Deep learning algorithms, for the classification of ASD vs typical subjects. They used public datasets such as ABIDE I and ABIDE II together with ADHD200.

 There are in my opinion some important issues:

 -the authors explicitly avoid to perform spatial normalization to a common template. The reason for this choice is that additional post processing steps may introduce biases.

However, a good spatial normalization is a powerful way to favor 3D algorithms convergence and to make importance features mapping interpretable.

Thank you for these comments on our approach. The goal of this paper is indeed to propose and provide an initial test of an alternative to using template registration when working with neurodivergent populations, where the risk of distorting data and introducing significant biases is higher. We agree that 3D algorithms may converge faster when all brains have the same shape and size. However, in our view, there is limited utility in having an algorithm converge well if the trade-off is the potential for distortion and biased metrics. In this work, we demonstrated the feasibility of an alternative pipeline that avoids this traditional preprocessing step. We do not claim that the proposed method is bias-free - in fact we emphasized the need to use multiple analytical algorithms, since each has its own biases.

In section 4.3. (p30), we added a discussion about the fact that classification was done in native space rather than template space, along with a proposal on how the cost or benefit of native space analysis could be measured in section 4.4. (p32).

Indeed, the authors process the gradCAM importance maps to be interpretable by adding an additional segmentation step and looking for correspondences between thresholded maps and segmentation which is quite complicated (and prone to biases such as those they tried to avoid).

We acknowledge that our interpretation approach may seem somewhat complicated, which is due to our goal of avoiding the spatial normalization step. A key step in our interpretation approach is the segmentation algorithm HighRes3DNet, a fast and readily implementable segmentation method available that avoids template registration. We combined this segmentation approach with guided GradCAM to enable interpretability. A similar interpretability analysis could be conducted on normalized brains, but for the reasons outlined above and in the paper, we opted to examine the feasibility of a pipeline that avoids normalization. Currently, there are limited alternatives to template registration for large-scale neuroimaging analyses. Our proof-of-concept work opens up possibilities for future research into developing and evaluating alternative pipelines that minimize potential sources of bias or distortion in research with neurodivergent populations.

We mentioned this point in our new discussion paragraph in section 4.4. (p32).

In addition, images acquired in the same site may be acquired with a standard positioning protocol which will be very peculiar for 3D classifier such those trained by the authors.

We are unsure about what the reviewer meant by this comment. To be clear about our methodology - our preprocessing of the raw 3D images was minimal, but did include an initial step in which each scan was reoriented to RAS+ orientation. This standardization of orientation was necessary so that all images used in training were consistent in terms of axes and orientation.

In the case of not well-balanced samples (for example with a predominance of ASD subjects in an acquisition site, this would imply a strong site biases which may be confused by an ASD effect.)

Thank you for raising this point. Indeed, we observed a multi-site effect, which could be attributed to several factors: differences in image acquisition protocols between sites (with the open question of which parameters have the biggest influence), varying levels of ASD severity across sites given the spectrum nature of ASD, differential sample size across sites (e.g., the algorithms were trained on 103 scans from NYU vs 5 from Stanford), unbalanced diagnosis/no-diagnosis representation within some sites (e.g. 27 ASD vs 76 non-ASD from NYU in the training set), comorbidities, gender and age distributions, and individual variability. Other potential contributors like geometrical factors related to the image data may also play a role.

We did not attempt to quantify the influence of these potential factors, since uneven sample sizes across sites posed a significant challenge and the meta-data available was insufficient. While we could have matched sample sizes across sites, this would have dramatically reduced the data set. We have added further discussion of these issues in section 4.3. (p30), acknowledging in section 4.4. (p32) that further investigations with larger, balanced datasets are needed to fully characterize the source(s) of site effects.

 -the performances on the test sets are very low and almost comparable with the chance level. Since the test sets are the only data on which a classifier demonstrates its capability to generalize, the throughout discussion on the importance features does not reflect the real brain areas by which the classifier is guided to distinguish ASD vs TD but the areas by which the classifier managed to optimize its performance on the training and validation data.

Thank you for your detailed feedback and the observations regarding the classifier's performance on the test sets. We agree that test sets are an important assessment of a model's capability to generalize. In our study, although test set performance could certainly be improved, it was consistent with that achieved by previous studies using Machine Learning models trained on sMRI data. It is important to note that we do not view this work as a final product but rather as an ongoing project that requires continuous improvement. As noted above, one goal was to establish the feasibility of a prediction pipeline that omitted spatial normalization. Our models’ comparable performance suggests that this is an analytic approach that is worth refining. We have openly shared all our code to foster collaboration, improvement, and reuse in other neuroimaging applications. We sincerely hope the community can build upon or adapt our work for refined applications.

We are unsure about what was meant by “*the importance features does not reflect the real brain areas by which the classifier is guided to distinguish ASD vs TD but the areas by which the classifier managed to optimize its performance on the training and validation data”*. We chose the guided grad-CAM algorithm (over grad-CAM) for its capability to provide class-specific output maps of voxel importance. We believe this is a useful way to identify and describe the anatomical brain patterns that contribute to accurate predictions of autism diagnosis (i.e., True Positives). Improving our model’s performance may result in changes to the set of regions identified, because it may open the model’s understanding to a wider range of data information, potentially comprising a greater variety of scanning protocols, better representativeness of the autism spectrum, etc.

-In S10 figure 8 it is clear that, despite the ASD gravity of deficit the performance of the classifier is completely dominated by the effect of site.

We agree that there is a clear site effect; we provided further evidence and illustration of this in S31 Table. However, it is important to note that S28, S29, S30 Figs represent just one diagnostic scale for ASD - SRS scores for social responsiveness. ASD diagnosis incorporates many other factors, such as those related to verbal/non-verbal communication scales, repetitive behavior metrics, etc. Examining algorithm performance across sites using these additional diagnostic dimensions may reveal better discrimination than the SRS scale alone and improve generalizability.

The multi-site prediction discrepancy highlights the ongoing challenge posed by site effects, which likely reflect multiple contributing factors, as discussed above. Further investigation is needed to elucidate the influence of heterogeneous scanning protocols, ASD subtypes/severity, age distributions, comorbidities, and other factors underlying these site effects. This represents an important future direction for developing more robust, widely applicable ASD prediction models. As mentioned above, we have now included additional consideration of these issues in the discussion section 4.3. (p30) and 4.4. (p32).

 -The authors did not show any MR brain images before and after brain extraction and intensity normalization. I suggest to show for at least 3 examples taken from different acquisition sites the raw images, the “brain extracted” images and the intensity normalized images to better understand the real input of the 3D deep classifiers.

Thank you for this suggestion; we have added an example demonstrating our preprocessing steps to the Supporting Information **S32 Fig**. Additionally, we have openly shared the full code for our preprocessing pipeline on GitHub for interested researchers who may wish to process their own neuroimaging data using our proposed methodology.

-The authors did not show any example of gradCAM maps and brain segmentation. This is important to understand how much focused are the maps of importance in comparison with the ROI segmentations.

Thank you for this suggestion - we have now added segmentation results from HighRes3DNet on the OASIS 0145 brain (referenced in the official documentation: https://github.com/fepegar/highresnet) to the Supporting Information **S33 Fig**. It is important to note that our interpretation pipeline utilized guided gradCAM to generate individual-specific maps of voxel importance. The maps are sparse but specific for each class (ASD/non-ASD). Since our goal was to identify replicable regions of importance at the cohort level, we performed a statistical analysis of relative voxel importance across all 1599 subjects. We have revised our description of the methodology and rationale in the methods section to ensure that these steps are clear and understandable to readers (see section 2.4, p11-15). In summary, guided gradCAM allowed sparse importance mapping at the level of the individual, but we leveraged these maps in aggregate to determine global trends, through statistical analysis of the relative frequencies of voxel importance.

-There are some parts in the results section for which the correspondent description in the methods section is missing (the effect of gender, the effect of age, the multi-site effect…).

Thank you for pointing out the need for more clarity in the paper. We have now revised the methods section 2.4.3 (p14-15) to ensure consistency.

-S1 table 1 and 2. Please reduce the significant figures.

Following your comment, we have done our best to reduce the corresponding tables.

 - S10 Fig 7, S10 Fig 8, and S10 Fig 9 please limit the x axis to 1.

Thank you for your comment. We regret that this was not possible because space was needed to include the legend for these figures.

We appreciate you taking the time to critically review our work - your feedback has helped improve the clarity and thoroughness of our description of our methodology.

Reviewer #2: This is a nicely written paper but the emphasis does not seem quite right. I am not a computational scientist but I do understand this technique in a broad way in line with PLOS 1's wide scope. So some issues may reflect my lack of knowledge on what is standard in the literature on machine learning.

 1, I understand this approach takes a lot of computational resources but computer time used is not the same burden as human time used. So I don't buy that saving processing time by not morphing the brains into MNI space is a major +. It makes more sense to do it for substantive reasons (i.e, capture anomalies about brain structure in ASD - but what? gyrification in temporal lobe?).

We appreciate you offering your perspective on the matter of computational efficiency. You raise a fair point that computer time is not equivalent to human time in terms of burden. Our intention was not to convey that efficiency alone was the prime goal, but rather to highlight it as an advantageous aspect of the proposed method, particularly when scaling to very large neuroimaging datasets, which is the direction of travel. In addition, the core aim of this work is not to propose a faster method but to present an *alternative* to template registration for neurodivergent populations, where the requirement to distort brains to fit with the “normal” or “standard” template (created using non-clinical populations) may negatively impact our ability to detect sample-related divergence in brain structure or function. We have modified our introduction to better emphasize this rationale (p4-6).

2. One take home I got was - these machine learning approaches on structural data including deep learning do not do a great job predicting autism. Site seems to matter - so maybe that is a real thing as culture does seem to affect behavioral differences in autism (koh & Milne).

You raise an excellent point - previous studies have demonstrated relatively poorer prediction performance for sMRI data compared to functional MRI data. However, research also indicates that combining sMRI and rs-fMRI modalities can further increase classification accuracy. This underscores the ongoing value of sMRI data, in conjunction with functional data, for elucidating brain-based autism biomarkers. While prior sMRI analyses relied on template registration, we present an alternative approach that avoids this step, as a starting point for developing new methodologies that may lead to further improvements in performance. Expanding this to encompass fMRI and multi-modal techniques is an important future direction.

As for cultural differences, we added a discussion in 4.3. (p30) on factors that may influence site-effects. We included the reference you provided (Koh & Milne, 2012).

3. A major plus is going back to identify the relevant structures. However,
 a. you do not give many references or ideas why these structures are important except for language, which is only true of some of them (for instance, TPJ = mentalizing), or whether/how they differ in ASD (based on references).

We chose not to discuss the regions in depth to avoid the risk of over-interpreting, since the results are somewhat unstable between the algorithms. Nonetheless, we have added further references to work that aligns with our findings and which generates suggestions for the potential direction of differences (raised in the next comment) in section 4.2 (p26-28).

b. you do not tell us how these structures differ in your data, but it seems that you cannot, right? COuld you test one theoretical prediction to examine this? If you have an idea of how they might differ (ie thicker frontal cortex).

This is an excellent point - working with raw, unprocessed data presents inherent challenges for interpreting the patterns captured by deep learning models. As discussed in Section 5.2., while DL can detect discriminative features, explaining the precise nature of those features and why they differ between populations requires additional investigations that are beyond the scope of this paper. In this work, we aimed to provide some initial interpretability insights by identifying brain regions deemed important by the models across demographics. We have openly shared code and results to enable further interrogation of the models and our findings by others in the field.

4. You present too many options for models, how to identify relevant areas etc. Decide which you think is (most) correct, and minimize discussion of the other options. Can you identify if the differences in specificity and sensitivity between models are significantly different and identify the best one? This also makes the figures too busy.

We appreciate this constructive feedback about the number of models examined in our study. The intention behind evaluating multiple models was to characterize the variability introduced by different architectures and to identify regions that were consistently identified as important for prediction across models, in order to account for model-specific biases. Our primary goal in this paper was to demonstrate the feasibility of a methodological approach. As above, we have modified the introduction to better clarify this rationale (p4-6).

5. A big question - why would you want to use expensive, hard to collect neuroimaging data to identify autism? you are likely to get better, cheaper predictive models from eye movements to a set of pictures or other behavioral measures. So I am not convinced we would ever use fMRI to identify autism, but if you can go back and identify the relevant regions and how they differ - that does seem useful.

We appreciate this question. We agree that it is not likely that neuroimaging data alone will diagnose autism - at least not soon or with our current technologies. Nonetheless, brain imaging *can* help to advance our understanding of the biology of Autism in ways that eye movements and behavioral data alone cannot. Using sophisticated data-driven techniques such as Deep Learning to identify/predict Autism using brain data can generate new leads about potential biomarkers which can then be pursued with more in-depth, hypothesis-driven approaches. Robust and reproducible biomarkers of Autism have proved elusive to date, which is why new methods, such as the approach we propose, which aims to avoid potential biases introduced by spatial normalization, are important.

6. Another major plus are the age and gender analyses. Perhaps expand a bit further, illustrate age, and clarify if they interact at all. why do predictive regions change with age? Are there typical changes in these age ranges that differ in ASD? of course more examination of the effect of IQ and comorbid conditions (like ADHD) would also be great.

Thank you for your comment. We fully appreciate the desire to explore these data further, but because the sample is highly imbalanced in terms of gender, because of the role of site effects discussed above, and given the modest model performance we do not want to risk overinterpretation. We have emphasized in the discussion (section 4.4; p32) that curating larger, better powered, and balanced datasets that would enable a thorough investigation of such effects should be a priority for the field.

7. I found these items jargony/ did not understand the concept fully:
 a. Guided Grad-CAM

Guided Grad-CAM is an algorithm that serves to understand what a CNN model looks at on the input image to return a given prediction. It is a combination of the guided backpropagation and the Grad-CAM algorithms. We have revised our explanation of the interpretation step in the methods section 2.4. (p11-15) to make it more accessible for readers.

b. not sure why you would ever use a 2-D program for 3-D brain data?

It is very common to use 2D algorithms when working with 3D data because working in 3 dimensions adds considerable complexity and computational load. As a result, few 3D algorithms exist. This is why we highlight the fact that we worked with 3-dimensional algorithms, since this enabled the algorithms to take the natural 3D structure of the images into account during processing. This is a novel aspect of our paper, relative to previous work.

c. would like more details in why gyrification differs in autism and how, since this is a good reason to not morph brain data (i.e., can change side of gyrus activation is evident on).

Thank you for your comment. We have added more detail regarding previous findings of differences in gyrification to the introduction (p4-6) to further justify our rationale for omitting the spatial normalization step.

d. differences/similarities between Machine learning vs Deep learning vs traditional multivariate approaches

We appreciate this comment and can understand the desire to better understand the differences between these approaches. We now succinctly explain the difference/similarities in the introduction (p5).

However, we are sure the reviewer can appreciate that providing more detailed explanations would require a considerable amount of text, which would distract from the main focus of the paper. To learn more, we would refer the reviewer to the book “The elements of statistical learning; second edition” (https://link.springer.com/book/10.1007/978-0-387-84858-7) and “Deep Learning” (https://www.deeplearningbook.org/).

e. not sure what cropping and padding is - just adding on to the edges?

Yes - as the reviewer suspects, cropping and padding simply modifies the field of view of an image by adding or removing lines to match a target shape. This transform modifies the affine matrix associated to the volume so that physical positions of the voxels are maintained (see https://torchio.readthedocs.io/transforms/preprocessing.html#croporpad).

f. I liked the discussion of false positives, negatives, etc but it gets a bit confusing as to the actual implications? Perhaps you could clarify with a table or just clarify writing?

Thank you for your suggestion. We have added explanations of the implications in sections 3.3.3. (p22-23) and 3.3.4. (p23).

Overall, we compared the results to check if the regions found important for False Positives were different or overlapped with the ones found important for True Positives. Idem for False Negatives and True Negatives.

g. section 4.4 does not make sense to me, in light of already using BET software (removing info outside the brain).

Yes, we extracted brains with BET. After this step, we normalized the voxel values across the entire image. As a result, the values of the voxels in the background are different from zero, and are not the same across participants. The CNN algorithm may take into account any value different from 0 during processing. Accordingly, it was important to examine whether image background was used in the predictions.

f. not sure what the inference step is?

Thanks for asking. The inference (or evaluation) step is a step after a DL model has been trained (i.e. optimization done), where we forward data through the DL neural network and get predicted outputs out of it.

Nice job in transparency and using large datasets.

Thank you very much for your review, comments and suggestions.

Reviewer #3: In summary, this manuscript aims to address the strong data heterogeneity and interpretability of deep learning in predicting Autism diagnosis based on structured MRI data and then proposes an interpretable predictive pipeline for inferring Autism diagnosis using 3D Deep Learning applied to minimally processed structural MRI scans. The design of the solution looks like reasonable and the analyses on experiments results are justified. However, the manuscript must be significantly revised and address the following issues.

1. The manuscript needs to be carefully revised from beginning to end. There are language errors and it needs to be polished by professional language experts.

Thank you for this feedback. We have now thoroughly and carefully revised and polished the paper from beginning to end.

2. The authors claim that one contribution of the study is the adoption of the minimal preprocessing pipeline , i.e. no transformation to template space, to avoid any impact of brain normalization on the detecting of Autism-related alterations in brain structure. However, the manuscript lacks a comparison with the relevant methods of using spatial template matching, which is not rigorous enough.

Thank you for raising this important point. Our primary aim was to introduce and demonstrate the feasibility of a new analytical approach that avoids template registration, rather than claim superiority over existing methods for this particular application. Rigorously benchmarking against traditional pipelines on large-scale data would require immense computing resources beyond our current capacity, particularly within the context of the large datasets examined. However, by making our code and models completely open source, we will enable the community to further evaluate and extend this method across diverse neuroimaging applications. Gradually accumulating insights from different groups applying this approach could ultimately provide a robust meta-analysis of its merits compared to established workflows.

We absolutely agree that as an initial foray, there is significant room for improving and optimizing our pipeline. Rather than position this as the definitive approach, our goal was to propose an alternative direction that avoids registration-induced distortion. Considerable refinement is needed, which can only be achieved through collaborative, iterative enhancement from the field.

We have tried to clarify this overall goal in the introduction (p4-7) and discussion (section 4.3; p30).

3. The resolution of the graphics in the current manuscript is too low, please replace it with high-quality graphics.

Thank you for your comment. Based on your recommendation, we have enhanced the resolution of the figures to the highest possible quality.

4. A large number of figures, tables, and experimental results are attached to the supporting material, which are important for method demonstration and should be included in the body, such as Table S5, S6, etc.

Thank you for your comment. We have made extensive use of supporting material in order to keep the paper concise and focused. We are concerned that moving any of these tables to the main manuscript will negatively impact the readability of the paper but are happy to defer to the editor on this point.

5. The proposed solution to the problem of data heterogeneity in the manuscript was achieved by training large data sets, which, in my opinion, did not completely or even alleviate the relevant problems, and therefore the description of this contribution should be removed. At the same time, to alleviate the problem of data heterogeneity, the author can explore the direction of reducing the distribution difference between the source domain and the target domain.

Thank you for this recommendation. We have highlighted that the challenge of heterogeneity remains to be resolved (p29-30). Indeed, reducing the distribution difference between the source domain and the target domain represents a future work direction, but is beyond the scope of the goals of the current paper. Our primary aim was to propose an alternative to normalization to template space. While normalization represents a way to reduce the variance of input data, it can introduce data distortion and greater bias in the case of neurodivergent populations. While we avoided normalization, we took other steps to reduce the variance of the input data - we applied minimal preprocessing transformations to reduce heterogeneity between the images (e.g., intensity normalization). We also restricted the training, validation, and first testing set to participants without comorbidities.

Addressing data heterogeneity is a key priority for future work. Much of this heterogeneity can be attributed to the broad spectrum of ASD diagnoses and the nuances of multi-site data collection. This includes variations in ASD type distributions among samples, differences in data acquisition protocols, and disparities in demographics and ethnicities across samples. Each aspect introduces numerous parameters that can influence the results, adding layers of complexity. Addressing the influence of these factors is an exciting area of research that promises insights into the biological bases of ASD through neuroimaging and, on a broader scale, a deeper understanding of psychiatric diagnoses.

We have added a discussion of this in section 4.3. (p30).

6. The performance of the autism diagnostic task in the manuscript was modest and lacked comparison with existing and conventional methods.

Thank you for your comment. We agree that the performance on the test set was modest. We briefly compare the performance with previous work (Sections 5.1 and 5.4), but also note that, to our knowledge, our paper is the first time that 3D-DL CNNs have been used to predict Autism diagnosis from 3D structural MRI scans without applying a brain normalization step.

Existing papers employed a variety of input data types and sample sizes, complicating direct comparisons. In addition to avoiding normalization to template, our primary aims were to evaluate the influence of CNN architecture on performance, the effects of including participants with and without comorbidities, and to provide a method for interpretability. Consequently, we concentrated on highlighting the unique contributions of our study and sharing our code and models for further exploitation, rather than comparing raw performance metrics with previous studies.

The open sharing of our code and pre-trained models has already resonated within the community. We have been approached by fellow researchers interested in building upon our work. While we believe there is room for further refinement and optimization of our method, its potential for replication across other diagnoses is evident—and may well already be in train.

Thank you very much for your review. We value your perspective and will keep it in mind as we continue our research and analysis in this area.

6. PLOS authors have the option to publish the peer review history of their article ([what does this mean?](https://journals.plos.org/plosone/s/editorial-and-peer-review-process#loc-peer-review-history)). If published, this will include your full peer review and any attached files.


 If you choose “no”, your identity will remain anonymous but your review may still be made public.


 **Do you want your identity to be public for this peer review?** For information about this choice, including consent withdrawal, please see our [Privacy Policy](https://www.plos.org/privacy-policy).

Reviewer #1: No

Reviewer #2: No

Reviewer #3: No

[NOTE: If reviewer comments were submitted as an attachment file, they will be attached to this email and accessible via the submission site. Please log into your account, locate the manuscript record, and check for the action link "View Attachments". If this link does not appear, there are no attachment files.]

 While revising your submission, please upload your figure files to the Preflight Analysis and Conversion Engine (PACE) digital diagnostic tool, <https://pacev2.apexcovantage.com/>. PACE helps ensure that figures meet PLOS requirements. To use PACE, you must first register as a user. Registration is free. Then, login and navigate to the UPLOAD tab, where you will find detailed instructions on how to use the tool. If you encounter any issues or have any questions when using PACE, please email PLOS at figures@plos.org. Please note that Supporting Information files do not need this step.
